# Femoral Nerve Palsy due to Anticoagulant Induced Retroperitoneal Hematoma

**DOI:** 10.1155/2014/450750

**Published:** 2014-10-16

**Authors:** Orcun Gurbuz, Abdulkadir Ercan, Gencehan Kumtepe, İlker Hasan Karal, Yusuf Velioglu, Serdar Ener

**Affiliations:** ^1^Department of Cardiovascular Surgery, School of Medicine, Balıkesir University, Bigadic, 10010 Balıkesir, Turkey; ^2^Department of Cardiovascular Surgery, Samsun Hospital for Education and Research, İlkadım, 55090 Samsun, Turkey; ^3^Department of Cardiovascular Surgery, Medical Park Usak Hospitals, 64000 Usak, Turkey; ^4^Department of Cardiovascular Surgery, Acıbadem Bursa Hospital, 16110 Bursa, Turkey

## Abstract

A forty-one-year-old man who, sought evaluation for a sudden hip flexion contracture and groin pain with a history of mechanical mitral valve replacement, had been misdiagnosed and treated as having lumbar discopathy for two days. This patient finally was diagnosed with compressive femoral neuropathy due to warfarin-induced retroperitoneal hematoma and successfully managed nonoperatively. This case is reported in order to draw attention to this rare presentation.

## 1. Background

The requirement for anticoagulation after valve surgery and difficulty of stabilizing the warfarin dosage can cause severe complications. Bleeding is known to be the main complication of oral anticoagulant therapy. It is classified as major if it is intracranial or retroperitoneal or leads to death or results in hospitalization or needs blood transfusion. The major bleeding rate was reported between 0.41% and 5.5% per year in several trials in patients receiving long-term oral anticoagulant therapy for prosthetic heart valves [1]. Spontaneous iliopsoas and retroperitoneal hematomas as a complication of anticoagulant therapy have been reported in various cases, with a relatively low incidence as 0.5% [2–5]. In the majority of reported cases, the diagnosis is often delayed as symptoms and signs are nonspecific. Therefore, retroperitoneal hematoma should be suspected in patients with significant groin, flank, abdominal, and back pain, partial loss of quadriceps functions, or haemodynamic instability in patients who are anticoagulated. Rarely, it can cause flexion contracture of the hip due to compressive femoral palsy as in the present case [3, 5]. There is no large series of patients to base decisions regarding treatment because of the rare presentation. In this case, patient managed conservatively with blood transfusion, rapid reversal of INR (international normalized ratio) to prevent further bleeding, and leg elevation to reduce neural compression.

## 2. Case Presentation

A 41-year-old man was admitted to the emergency department for left hip flexion contracture and severe groin pain. He had been complaining of progressive lower abdomen and groin pain, paraesthesia, and weakness in his upper left thigh for three days. Therefore, this case was misdiagnosed and treated as lumbar discopathy previously in another hospital two days ago. He had a history of prosthetic mitral valve replacement (MVR) two years ago and was taking warfarin. Antiplatelet therapy (acetylsalicylic acid at 100 mg/day) was added to the medical therapy three months ago by a cardiologist from another hospital.

**Figure 1:**
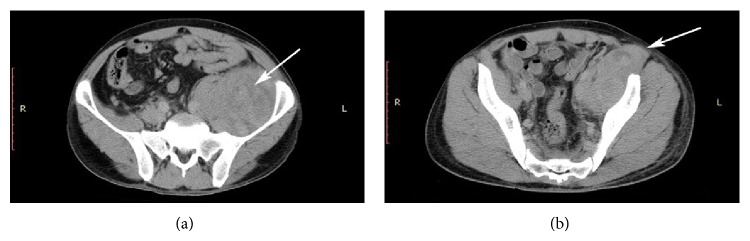
Abdominal CT showed a large retroperitoneal hematoma, which extends through femoral triangle. White arrows in the pictures highlight left iliopsoas hematoma and its inguinal extension.

On physical examination, he presented with a fixed left hip in semiflexion (about 45°) and any further passive movement of the hip was giving him pain in the groin. He had a good general status, with blood pressure of 100/60 mm Hg, pulse 96 beats*·*min^−1^, respiratory rate 14 breaths*·*min^−1^, and an axillary temperature of 36.5°C. Both lower extremity pulses were symmetric and equal. Laboratory test revealed anaemia with a haemoglobin value of 8.9 g/L. The INR level was 4.1 and the prothrombin time (PT) was 45.1 seconds. Ultrasonography (US) detected 10 × 8 × 8 cm left iliopsoas hematoma. This finding was confirmed by computed tomography (CT) (Figures 1(a) and 1(b)).

**Figure 2:**
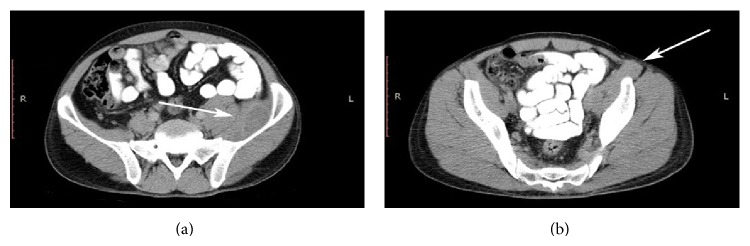
Repeat CT sections after 3 weeks showed significant reduction of hematoma. White arrows in the pictures highlight left iliopsoas hematomas reduction and the reduction of its inguinal extension.

We preferred conservative management due to stable parameters with careful hemodynamic and haemoglobin level monitoring. Right leg was kept elevated above the heart level to reduce nerve compression by hematoma. Warfarin and antiplatelet therapies were withdrawn. The INR was lowered to 1.9 within 24 hours after the administration of 10 mg vitamin K and 2 fresh frozen plasma units, so low molecular weight heparin (LMWH) therapy started. One hematite concentrate was administered in order to recover normal values. Flexion contracture recovered within 24 hours and left quadriceps strength improved to 3/5 and the patient began mobilization. Haemoglobin level did not reduce and hemodynamic parameters remained stable during hospitalization. A follow-up ultrasound scan was performed after 72 hours, and no growth was seen in the hematoma. On the third day, haemoglobin level was 9.9 g/L and INR was 1.32. The quadriceps strengthening exercise program was started on day three of hospitalization after being sure that bleeding ceased. The patient was discharged on the sixth day with normal laboratory findings and 3/5 strength in his left quadriceps. On his 21st day visit after hospitalization, strength of left quadriceps femoris was 4/5, haemoglobin level was 12.2 g/L, and US scan revealed a decrease in hematoma (3.5 × 2.5 cm). Abdominal CT confirmed partial resolution of the hematoma (Figures 2(a) and 2(b)). A single warfarin regimen without antiplatelet therapy was initiated after these findings. LMWH continued until the INR reached 2.5. All symptoms resolved during the next month, and the patient's sensation, strength, and reflexes returned to normal.

## 3. Conclusions

Retroperitoneal haematoma incidence in patients undergoing therapeutic anticoagulation varies between 0.6% and 6.6% among studies [1, 6]. The intensity of anticoagulant effect appears to be the most important risk factor for major hemorrhage, independent of the indication for therapy; all randomized controlled trials have reported a strong relationship between the targeted intensity of anticoagulant therapy and the risk of major bleeding [1, 7]. The optimal therapeutic range of anticoagulant therapy in the secondary prevention of vascular events lies between INRs from 2.0 to 3.9, with a target INR of 3.0. When the INR is above 5.0, the risk of serious bleeding complications becomes unacceptable [7]. Moreover, it has also been shown that adding antiplatelet medications to warfarin therapy significantly increases the incidence of major bleeding [1]. Accordingly, in this case, hematoma occurred after antiplatelet therapy had been started, but when INR had just reached over the therapeutic range.

The femoral nerve provides motor innervation to the quadriceps, sartorius, pectins, and iliopsoas muscle and supplies sensory innervation to the anteromedial thigh and medial leg. It lies between the iliacus and psoas muscles, which form a tendon inserting into the lesser trochanter of the femur. The entrapment of the femoral nerve in this area due to hematoma causes weakness in hip flexion and knee extension and has been reported more widely after hematologic disorders such as haemophilia [8, 9] and leukaemia [10]; other causes are uncommon [2–5, 11–14]. Some of the sudden symptoms such as lateral abdominal pain, hemiparesis, or hip flexion contractures are nonspecific and can refer to other diseases. Diagnosing such cases may be challenging for clinicians in the hemodynamically stable patient, especially when the INR level is not too high, as our patient has experienced. Therefore, the clinician should have a high index of suspicion and need to be aware of this rare presentation. An abdominal ultrasound can satisfactorily detect retroperitoneal hematoma; this will reduce morbidity and ameliorate recovery.

The treatment of spontaneous retroperitoneal hematomas causing femoral neuropathy remains controversial. Favourable results have been reported with conservative therapy, in hematological pathology [2–5, 8–10], but, in the case of progressive neurological dysfunction, urgent operative intervention has been suggested, especially in cases without disorders of haemostasis [11, 12]. In our case, we chose conservative therapy with close hemodynamic and neurological monitoring. Furthermore, the affected extremity was kept elevated for 2 days. Flexion contracture recovered rapidly within 24 hours, and the patient began mobilization. We think that rapid recovery of hip flexion contracture might have been caused by the elevation of the extremity by decreasing the compression of the nerve in the groin. Following an acute retroperitoneal hematoma, bleeding should be collected in the pelvic region due to gravity and it might compress the femoral nerve if it extends into the inguinal canal. In such cases, elevation of the affected extremity against gravity would direct the bleeding upwards and decompress the nerve.

In conclusion, in the presence of combined anticoagulant and antiplatelet therapy, iliopsoas hematoma may occur without a too high INR level. For every anticoagulated patient suffering from lower abdominal and pelvic pain, abdominal ultrasound or CT scanning might be performed to make an accurate diagnosis. Medical treatment can be combined with elevation of the affected extremity to decompress the femoral nerve entrapment.

## Abbreviations

CT: Computed tomography
INR: International normalized ratio
LMWH: Low molecular weight heparin
MVR: Mitral valve replacement
PT: Prothrombin time
US: Ultrasonography.
